# Supplementary material for: Room-Temperature Solution-Processed 0D/1D Bilayer Electrodes for Translucent CsPbBr3 Perovskite Photovoltaics
Source: Nanomaterials (Basel). 2021 Jun 4;11(6):1489. doi: 10.3390/nano11061489 (PMC8227939; doi:10.3390/nano11061489)
Supplement: Supplementary file 1 [file nanomaterials-11-01489-s001.zip › nanomaterials-1239344-supplementary.pdf]

Supplementary Materials

Room Temperature Solution Processed 0D/1D Bilayer Electrodes for translucent $CsPbBr_3$ Perovskite Photovoltaics

Bhaskar Parida[1], Saemon Yoon[1] and Dong-Won Kang[1,*]

[1] School of Energy Systems Engineering, Chung-Ang University, Seoul 06974, Republic of Korea.

* Correspondence: kangdwnl@cau.ac.kr; Tel.: +82-2-820-5414

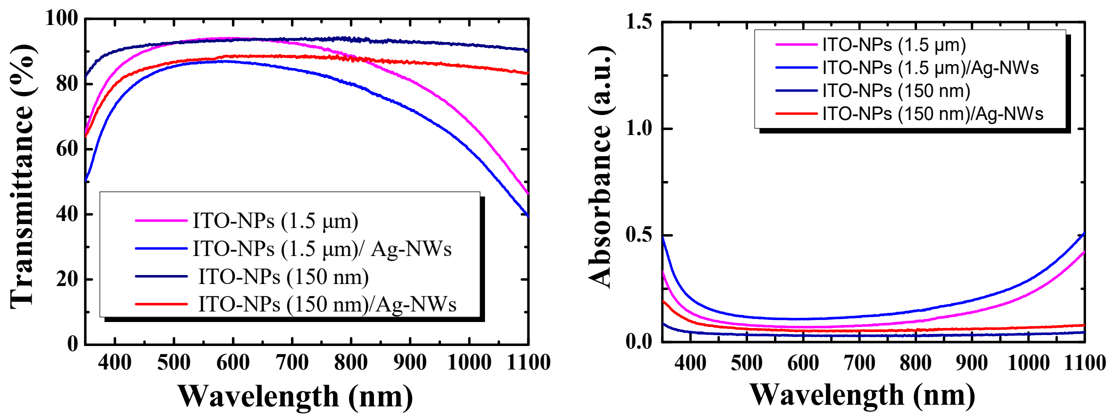

Figure S1. The optical transmittance and absorption of the ITO-NPs, ITO-NPs/Ag-NWs samples with different thickness in ITO-NPs (150 nm and 1.5 μm)

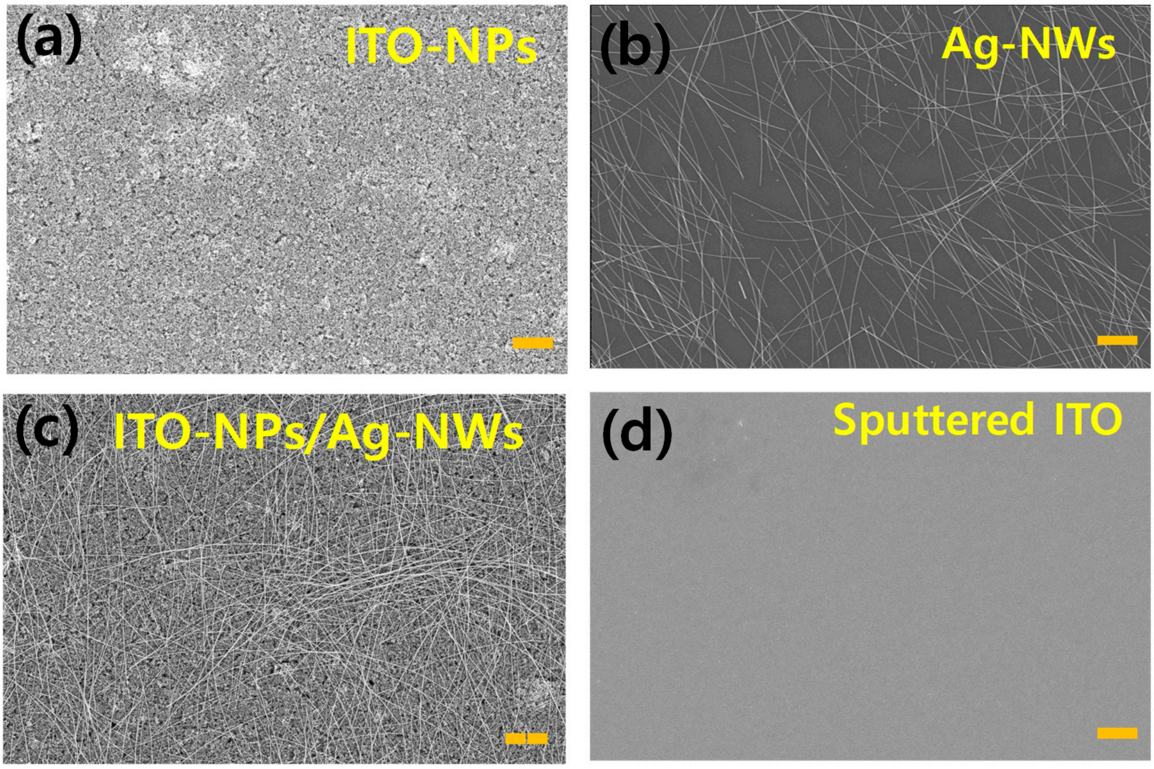

Figure S2. Field enhanced scanning electron microscope (FE-SEM) top view images of ITO-NPs, Ag-NWs, ITO-NPs/Ag-NWs bilayer, and sputtered ITO films (2 μm in scale bar).

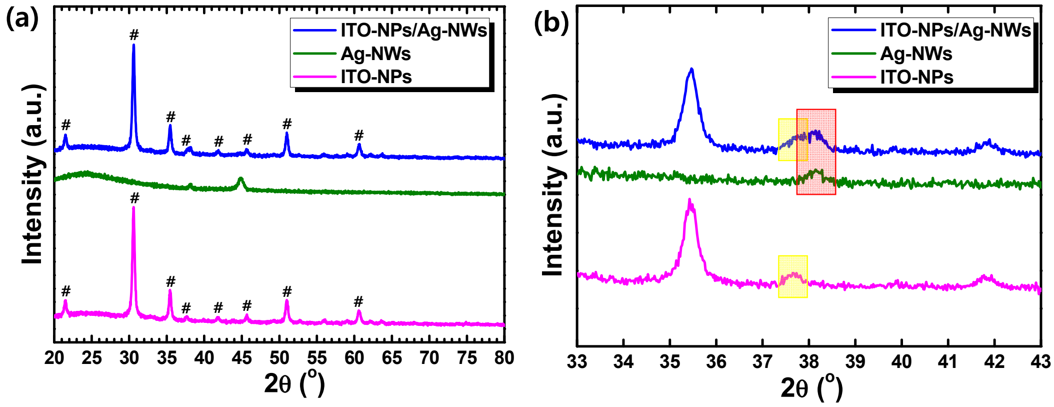

Figure S3. (a) XRD patterns for comparison of ITO diffraction peaks (symbol : #) and (b) the enlarged comparison of ITO-NPs (yellow rectangle) and Ag-NWs (red rectangle) diffraction peaks with ITO-NPs, Ag-NWs and ITO-NPs/Ag-NWs films.

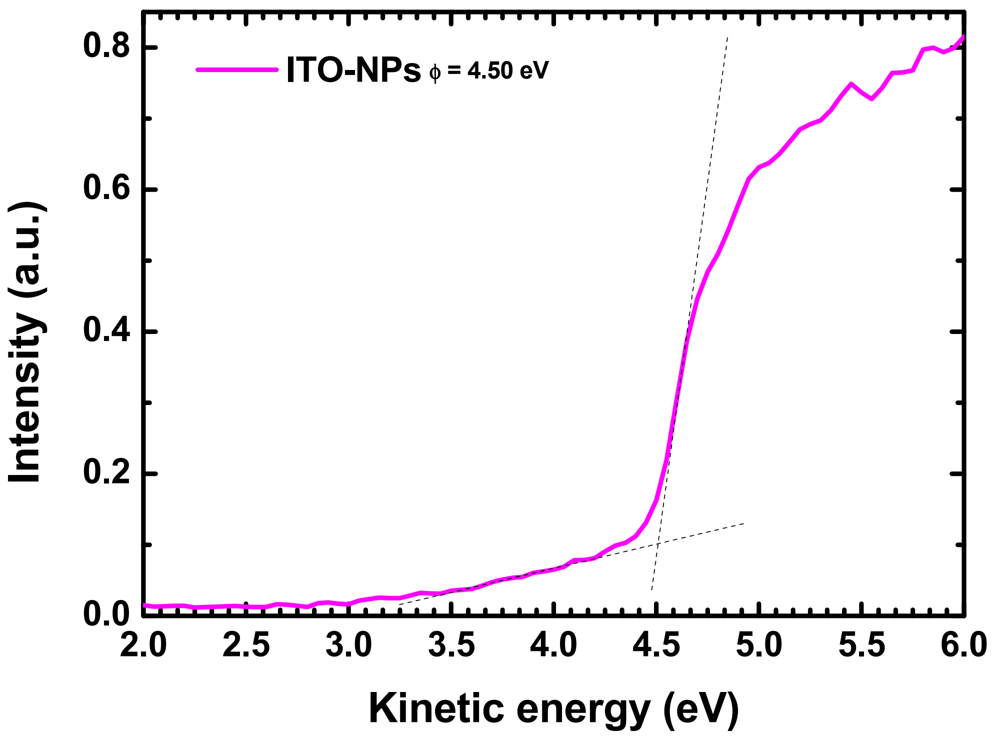

Figure S4. UPS spectra of ITO-NPs films.

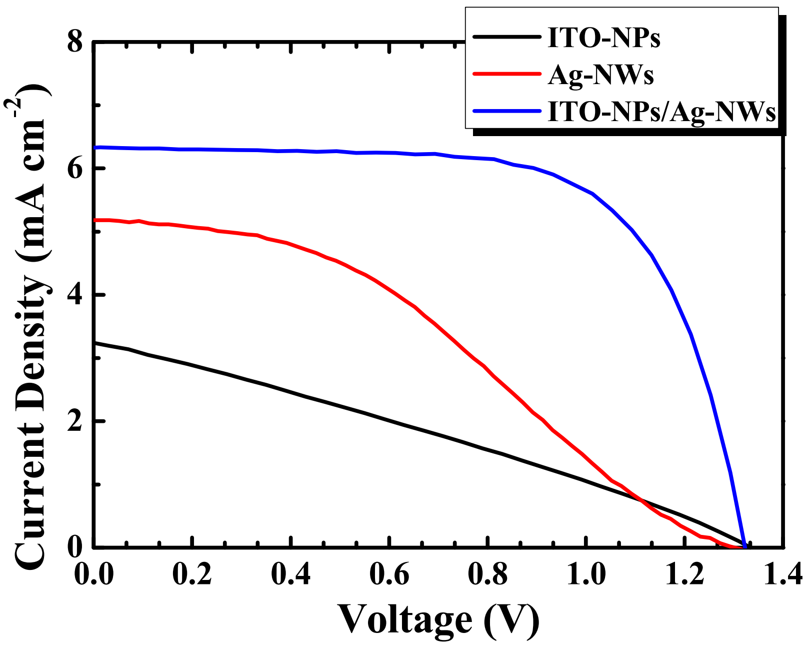

Figure S5. J-V properties of fabricated $CsPbBr_3$ perovskite solar cells with various solution-processed TEs.

Table S1. Device performance of fabricated $CsPbBr_3$ perovskite solar cells with various solution-processed TEs.

Electrodes	V_{OC} (V)	J_{SC} (mA cm^{-2})	FF	PCE (%)
ITO-NPs	1.33	3.20	0.299	1.27
Ag-NWs	1.29	5.18	0.370	2.48
ITO-NPs/Ag-NWs	1.32	6.33	0.677	5.64

Table S2. The detail PV parameters with statistical data for fabricated $CsPbBr_3$ solar cells with various rear electrode were included.

Electrodes	V_{OC} (V)	J_{SC} (mA cm^{-2})	FF	PCE (%)
Au (Opaque)	1.29 (±0.04)	6.84 (±0.35)	0.697 (±0.031)	6.16 (±0.43)
ITO (Vacuum)	0.82 (±0.22)	6.19 (±0.25)	0.421 (±0.100)	2.26 (±1.01)
ITO-NPs/Ag-NWs (Solution)	1.30 (±0.03)	6.19 (±0.27)	0.627 (±0.044)	5.06 (±0.39)
